# Prediction of school outcome after preterm birth: a cohort study

**DOI:** 10.1136/archdischild-2018-315441

**Published:** 2018-10-08

**Authors:** David Odd, David Evans, Alan M Emond

**Affiliations:** 1 Neonatal Unit, North Bristol NHS Trust, Bristol, UK; 2 Population Health Sciences, Bristol Medical School, University of Bristol, Bristol, UK

**Keywords:** premature birth, preterm, cohort studies, ALSPAC, education

## Abstract

**Objective:**

To identify if the educational trajectories of preterm infants differ from those of their term peers.

**Design:**

This work is based on the Avon Longitudinal Study of Parents and Children (ALSPAC). Educational measures were categorised into 10 deciles to allow comparison of measures across time periods. Gestational age was categorised as preterm (23–36 weeks) or term (37–42 weeks). Multilevel mixed-effects linear regression models were derived to examine the trajectories of decile scores across the study period. Gestational group was added as an interaction term to assess if the trajectory between educational measures varied between preterm and term infants. Adjustment for possible confounders was performed.

**Subjects:**

The final dataset contained information on 12 586 infants born alive at between 23 weeks and 42 weeks of gestation.

**Main outcome measures:**

UK mandatory educational assessments (SATs) scores throughout educational journal (including final GCSE results at 16 years of age).

**Results:**

Preterm infants had on average lower Key Stage (KS) scores than term children (−0.46 (−0.84 to −0.07)). However, on average, they gained on their term peers in each progressive measure (0.10 (0.01 to 0.19)), suggesting ‘catch up’ during the first few years at school. Preterm infants appeared to exhibit the increase in decile scores mostly between KS1 and KS2 (p=0.005) and little between KS2 and KS3 (p=0.182) or KS3 and KS4 (p=0.149).

**Conclusions:**

This work further emphasises the importance of early schooling and environment in these infants and suggests that support, long after the premature birth, may have additional benefits.

**What is already known on this topic?**

- Preterm birth is a relatively common event, with 6% of infants being born 4 or more weeks before their due date.
- Ex-preterm children are more likely to struggle at school than term peers.
- It is unclear if preterm infants demonstrate ‘catch up’ or begin to struggle more as they grow.

**What this study adds?**

- Early educational measures are correlated with later measures.
- The trajectory of educational measures in preterm infants varies compared with that of their term peers.
- Most of the ‘catch up’ seems to occur in the first few years at school.

## Introduction

Preterm birth is a relatively common event, with 6% of infants being born 4 or more weeks before their due date.^1^ However, both extreme preterm birth and less severe prematurity carries a higher risk of mortality,^2^ and long-term cognitive,^3^ educational,^4^ psychiatric^4^ and social impacts^5^ for the infant. We have recently shown that preterm infants were more likely to struggle at school,^1 6^ especially those enrolled in school a year earlier due to their prematurity. However, while this effect was measurable throughout their educational journey (up to the age of 16), it is unclear if preterm infants demonstrate ‘catch up’ as they grow,^7^ or alternatively begin to struggle more as the demands on them become more complex. The primary aim of this work is to identify if early educational measures are more, or less, predictive of final attainment in preterm infants than term infants, and if the educational trajectories of preterm infants differ from those of their term peers.

## Methods

Avon Longitudinal Study of Parents and Children (ALSPAC) recruited 14 541 pregnant women resident in Avon, UK, with expected dates of delivery from 1 April 1991 to 31 December 1992.^8 9^ Briefly, 14 541 pregnancies were initially enrolled. Of these initial pregnancies, there was a total of 14 062 live births and 13 988 children who were alive at 1 year of age. More information can be found on the ALSPAC website: www.alspac.bristol.ac.uk.

Outcome measures for this work were derived from the routine educational assessments mandatory in state schools in England which were linked to the ALSPAC study. In England, a child’s educational journey at school is split into four ‘Key Stages’, with assessments at the end of each stage: Key Stage 1 (KS1) (ages 5–7 years), Key Stage 2 (KS2) (ages 7–11 years), Key Stage 3 (KS3) (ages 11–14 years) and Key Stage 4 (KS4) (ages 14–16 years). For the predictive models, a poor outcome at age 16 (KS4) was defined as not obtaining 5 GCSE passes at A* to C level. This is consistent with our previous work and provide a more meaningful measure for interpretation.^6^ Where comparisons across KSs were needed, the summary measures were categorised into 10 deciles to allow comparison of measures across time periods. Gestational age at birth was prospectively recorded from the clinical notes and if less than 37 weeks was then confirmed after reviewing the clinical records. Gestational age was categorised as preterm (23^+0^ to 36^+6^ weeks) or term (37^+0^ to 42^+6^ weeks).

Potential confounders between gestation at birth and educational outcome were identified a priori^10^ and split into three groups:

- Social factors: maternal age, socioeconomic group^11^ and education and ethnicity.
- Antenatal factors: gender, parity, weight, length and head circumference at birth.
- Intrapartum factors: mode of delivery and maternal hypertension.

The dataset contained information on 13 991 infants born alive at between 23 weeks and 42 weeks of gestation. Infants were defined as preterm (n=898) or term (n=13 093). A total of 1405 infants did not have outcome measures available, leaving 12 586 infants. The dataset used has been described in our previous work,^6^ but in brief, infants excluded from the analysis were more likely to have older mothers, with higher socioeconomic status and more educational qualifications. The excluded infants were more likely to be male, had lower Apgar scores and were more likely to have received resuscitation at birth.

Initially, the demographics of the population, split by gestational age category, were described, and then correlations between the 10 KS deciles were derived, and the proportions of infants having a low score at each measure were assessed. The proportion of infants scoring each combination of KS1 and KS4 deciles was then plotted, split by gestational status.

A multiple imputation data technique (chained equations) was used to minimise any potential selection bias in the multivariable models (below) and to facilitate reporting on the same number of subjects for crude and adjusted analyses.^12^ These models were derived using all the variables presented in this paper (including exposure and outcome variables). Analysis was limited to infants with gestational age and the appropriate outcome measure (ie, imputed outcome values were not used).

Receiver operating characteristic (ROC) curves were then produced to investigate how well KS1 scores could predict a low KS4 score, and if gestational age modified the relationship. Adjustment for possible confounders was next performed by adding the potential confounders to the regression models, in the blocks of common variables defined above (eg, social factors). The model was then repeated using KS2 and KS3 measures instead of KS1.

Finally, multilevel mixed-effects linear regression models were then derived to examine the trajectories of decile scores across the study period. Dependent variables were the KS decile, while explanatory measures were age, preterm status and other covariates (see above). Data were treated as clustered by child, and overall linear changes between KS measures were assessed using the Stata command ‘xtmixed’. Gestational group was added as an interaction term to assess if the trajectory between educational measures varied between preterm and term infants. Adjustment for possible confounders was performed as above. In a sensitivity analysis, this model was repeated to assess if the results were attenuated after adjustment for infants in receipt of special educational needs support. In a final sensitivity analysis, the model was repeated and tested, including only preterm infants, to test if the trajectory of their educational performance was modified by whether they were in the correct school year (due to a discrepancy between their estimated date of delivery and actual date of birth) or not.

All analyses were conducted with Stata V.14. Results are presented as OR (95% CI), mean (SD), median (IQR) or number (%).

## Results

The cohort is drawn from the ALSPAC cohort and is identical to our previous work.^6^ The median gestation was 35 (IQR 33–36) weeks in the preterm group and 40 (IQR 39–41) in the term group. Demographics of the infants are shown in table 1. Preterm infants varied from term infants in a number of ways. Of note, they had lower birth weights, lengths and head circumferences, lower Apgar scores and were more likely to be born as multiple births. The distributions of four KS scores, overlaid with the derived deciles (and the mean score per decile), are shown in the online supplementary appendix.

10.1136/archdischild-2018-315441.supp1 Supplementary file 1

**Table 1:** Characteristics of study population

Measure	Number with data	Preterm (n=775)	Term (n=11 811)	P values
Prepregnancy factors				
Maternal age	12 586	27.5 (4.9)	27.9 (5.0)	0.0247
Maternal socioeconomic group	9052			0.930
I—Professional		22 (4.3%)	460 (5.5%)	
Ii—Managerial		158 (31.0%)	2610 (31.0%)	
iiiN—Skilled non-manual		41 (8.1%)	685 (8.0%)	
iiiM—Skilled manual		228 (44.8%)	3729 (43.7%)	
iv—Semiskilled		49 (9.6%)	863 (10.1%)	
v—Unskilled		11 (2.2%)	196 (2.3%)	
Mother’s highest educational qualification*	11 175			0.005
CSE		170 (26.4%)	2182 (20.7%)	
Vocational		70 (10.9%)	1079 (10.2%)	
O level		205 (31.9%)	3730 (35.4%)	
A level		137 (21.3%)	2291 (21.8%)	
Degree		61 (9.5%)	1250 (11.9%)	
Non-white ethnicity		66 (9.3%)	488 (4.5%)	<0.001
Antenatal and intrapartum factors				
Primiparous	11 632	348 (48.7%)	4804 (44.0%)	0.227
Maternal hypertension	12 585	105 (13.6%)	406 (3.4%)	<0.001
Multiple birth	12 586	149 (19.2%)	186 (1.6%)	<0.001
Delivery	11 465			<0.001
Spontaneous cephalic		427 (58.3%)	8191 (76.3%)	
Emergency caesarean section		166 (22.7%)	624 (5.8%)	
Elective caesarean section		40 (5.5%)	449 (4.2%)	
Instrumental		62 (8.5%)	1323 (12.3%)	
Breech		37 (5.1%)	146 (1.4%)	
Infant and postpartum factors				
Male	12 586	443 (57.2%)	6033 (51.1%)	0.001
Birth weight (g)	12 441	2347 (615)	3456 (485)	<0.001
Birth length (cm)	9518	47.0 (2.6)	50.8 (2.3)	<0.001
Head circumference (cm)	9664	32.4 (2.1)	34.9 (1.4)	<0.001
Apgar at 1 min	11 467	9 (7–9)	9 (8–9)	<0.001
Apgar at 5 min	11 467	9 (9–10)	10 (9–10)	<0.001
Received resuscitation	11 452	182 (24.9%)	838 (7.8%)	<0.001

SD are given for means of normally distributed continuous variables and percentages for proportions.

*CSE=Certificate in Secondary Education (commonly taken at 16 years of age); Vocational=City & Guilds (intermediate level), technical, shorthand or typing, or other qualification; O level=Ordinary level (commonly taken at 16 years of age); A level=Advanced level (commonly taken at 18 years of age), state enrolled nurse, state registered nurse, City & Guilds (final or full level) or teaching qualification; Degree=University degree.

**Figure 1:**
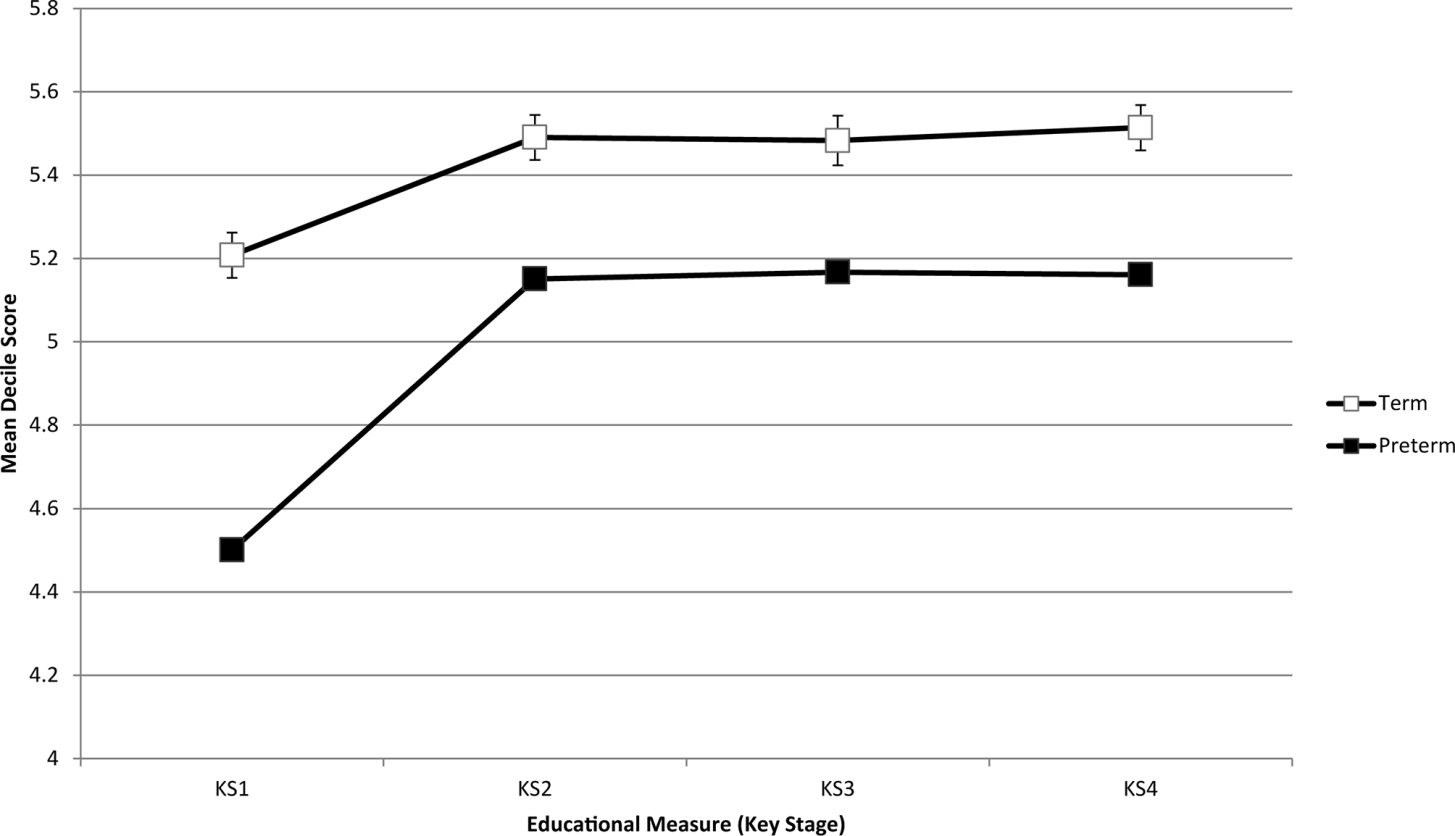
Summary measures of Key Stage scores at each time point, split by gestational age groups.

Preterm infants had a higher chance of being in the lowest decile at all four assessments than their term peers (KS1: 139 (17.9%) vs 1310 (11.1%), p<0.001; KS2 91 (11.7%) vs 1079 (9.1%), p=0.015; KS3 74 (9.6%) vs 875 (7.4%), p=0.029, KS4 102 (12.2%) vs 1039 (8.8%), p<0.001). Correlations of low KS scores were attenuated by the length of time that passed between the two assessments (table 2), with the highest correlation being between a KS2 and KS3 measure (0.81 (0.81–0.82)) and the lowest between a KS1 and KS4 measure (0.63 (0.61–0.64)). Figure 1 shows the summary measures of KS scores at each time point, split by gestational age.

**Table 2:** Correlations between KS deciles

	KS1	KS2	KS3	KS4
KS1	1			
KS2	0.78 (0.78 to 0.79)	1		
KS3	0.67 (0.65 to 0.68)	0.81 (0.81 to 0.82)	1	
KS4	0.63 (0.61 to 0.64)	0.73 (0.72 to 0.74)	0.73 (0.72 to 0.74)	1

Numbers are correlation coefficients (95% CI).

All p values <0.001.

KS, Key Stage.

ROC curves showed that the mean area under the curve for predicting a low score at KS4 ranged from 0.83 (0.82–0.84) in the model only containing KS1 to 0.89 (0.88–0.89) in the saturated model using KS2 as the educational measure (table 3). There was little overall evidence that preterm status modified the predictive value of KS 1, 2 or 3 deciles on a final low KS4 score.

**Table 3:** ROC curve analysis for the prediction of a poor KS4 score

Educational measures included	Unadjusted		Adjusted for social factors*		Adjusted for social* and antenatal factors†		Adjusted for social*, antenatal† and intrapartum‡ factors	
	AUC	P_interaction_	AUC	P_interaction_	AUC	P_interaction_	AUC	P_interaction_
KS1	0.83 (0.82 to 0.84)	0.274	0.85 (0.84 to 0.86)	0.339	0.85 (0.84 to 0.86)	0.278	0.85 (0.84 to 0.86)	0.275
KS2	0.87 (0.87 to 0.88)	0.776	0.88 (0.88 to 0.89)	0.675	0.89 (0.88 to 0.89)	0.621	0.89 (0.88 to 0.89)	0.647
KS3	0.87 (0.86 to 0.87)	0.213	0.87 (0.87 to 0.88)	0.173	0.88 (0.87 to 0.88)	0.175	0.88 (0.87 to 0.88)	0.157

Outcome is the area under the ROC curves. P values are for interaction with preterm status.

*Social factors: maternal age, socioeconomic group and education and ethnicity.

†Antenatal factors: gender, parity, weight, length and head circumference at birth.

‡Intrapartum factors: mode of delivery and maternal hypertension.

AUC, area under the curve; KS, Key Stage; ROC, receiver operating characteristics.

Table 4 shows summary results from the mixed linear model. Over the four measures, preterm infants tended a have a mean decile score around 0.5 lower than term infants (−0.46 (−0.84 to −0.07)). There was also strong evidence that preterm infants gained around a tenth of a decile on their term peers in each progressive measure (0.10 (0.01 to 0.19)), suggesting that preterm infants exhibited a different trajectory to term infants. When looking at the difference between each measure, preterm infants appeared to exhibit the increase in decile scores mostly between KS1 and KS2 (0.34 (0.10 to 0.58)) and little between KS2 and KS3 (p=0.182) or between KS3 and KS4 (p=0.149). Adding the variable of special educational needs support to the model produced compatible results to the main analysis (trajectory difference 0.11 (0.01 to 0.20), p=0.025). In a model containing just preterm infants, there was little evidence that infants placed in the incorrect school year due to their prematurity had a different profile of ‘catch up’ in their deciles than those in the correct school year (p=0.130).

**Table 4:** Mixed linear regression models for the mean KS decile difference between preterm and term infants

Covariate	Unadjusted		Adjusted for social factors*		Adjusted for social* and antenatal factors†		Adjusted for social*, antenatal† and intrapartum‡ factors	
	Mean difference (95% CI)	P values	Mean difference (95% CI)	P values	Mean difference (95% CI)	P values	Mean difference (95% CI)	P values
Mean difference in score	−0.77 (–1.01 to −0.52)	<0.001	−0.49 (–0.80 to −0.18)	0.002	−0.59 (–0.89 to −0.13)	0.009	−0.46 (–0.84 to −0.07)	0.021
Change over four measures								
Overall (KS1 to KS4)	0.10 (0.04 to 0.17)	0.001	0.10 (0.02 to 0.17)	0.017	0.10 (0.01 to 0.19)	0.035	0.10 (0.01 to 0.19)	0.035
KS1 to KS2	0.24 (–0.09 to 0.40)	0.002	0.28 (–0.08 to 0.47)	0.006	0.34 (0.10 to 0.58)	0.005	0.34 (0.10 to 0.58)	0.005
KS2 to KS3	−0.02 (–0.16 to 0.12)	0.785	−0.02 (–0.20 to 0.16)	0.830	−0.14 (–0.35 to 0.07)	0.188	−0.15 (–0.36 to 0.07)	0.182
KS3 to KS4	0.13 (–0.04 to 0.30)	0.125	0.08 (–0.14 to 0.29)	0.496	0.18 (–0.07 to 0.44)	0.166	0.19 (–0.07 to 0.45)	0.149

Measures are mean differences (95% CI) in the average KS decile and in the change seen over and between the four measures.

*Social factors: maternal age, socioeconomic group and education and ethnicity.

†Antenatal factors: gender, parity, weight, length and head circumference at birth.

‡Intrapartum factors: mode of delivery and maternal hypertension.

KS, Key Stage.

Finally, the analysis was repeated, splitting the preterm cohort by those born extremely preterm (23^+0^ to 31^+6^ weeks of gestation, n=101), and those moderate/late preterm (32^+0^ to 36^+6^ weeks of gestation, n=674) (table 5). For the moderate/late preterm infants, results were entirely compatible with the main analysis. For the extremely preterm infants, overall trajectory was similar to the main analysis (0.11 (−0.01 to 0.23)), although there was some evidence that some of the gains seen between KS1 and KS2 (0.50 (0.19 to 0.82)) were lost between KS2 and KS3 (−0.35 (−0.62 to −0.07)). However, small numbers and wide CIs limit interpretation. There was little evidence that overall the trajectory of educational deciles was different between the two preterm groups (p=0.365).

**Table 5:** Mixed linear regression models for the mean KS decile difference between extreme preterm or moderate/late preterm birth and term infants

Covariate	Unadjusted		Adjusted for social factors*		Adjusted for social* and antenatal factors†		Adjusted for social*, antenatal† and intrapartum‡ factors	
	Mean difference (95% CI)	P values	Mean difference (95% CI)	P values	Mean difference (95% CI)	P values	Mean difference (95% CI)	P values
Extreme preterm infants								
Mean difference in score	−0.70 (–1.04 to −0.37)	<0.001	−0.50 (–0.93 to −0.06)	0.025	0.45 (–0.95 to 0.05)	0.080	−0.40 (–0.91 to 0.11)	0.122
Changes over four measures								
Overall (KS1 to KS4)	0.09 (0.01 to 0.18)	0.030	0.09 (−0.01 to 0.20)	0.087	0.11 (–0.01 to 0.23)	0.075	0.11 (–0.01 to 0.23)	0.072
KS1 to KS2	0.17 (–0.04 to 0.38)	0.121	0.34 (0.07 to 0.61)	0.015	0.50 (0.19 to 0.81)	0.002	0.50 (0.19 to 0.82)	0.002
KS2 to KS3	−0.05 (–0.25 to 0.15)	0.615	−0.14 (–0.38 to 0.11)	0.271	−0.34 (–0.61 to −0.07)	0.015	−0.35 (–0.62 to −0.07)	0.015
KS3 to KS4	0.21 (–0.03 to 0.44)	0.081	0.17 (–0.13 to 0.47)	0.263	0.32 (–0.02 to 0.65)	0.062	0.33 (–0.01 to 0.67)	0.060
Moderate or late preterm								
Mean difference in score	−0.63 (–0.89 to −0.37)	<0.001	−0.42 (–0.74 to −0.10)	0.011	−0.47 (–0.86 to 0.09)	0.016	−0.42 (–0.80 to −0.03)	0.036
Changes over four measures								
Overall (KS1 to KS4)	0.10 (0.03 to 0.16)	0.004	0.09 (0.01 to 0.17)	0.031	0.10 (0.01 to 0.20)	0.029	0.11 (0.01 to 0.20)	0.028
KS1 to KS2	0.25 (0.09 to 0.41)	0.003	0.27 (0.07 to 0.48)	0.009	0.34 (0.10 to 0.58)	0.006	0.33 (0.10 to 0.59)	0.006
KS2 to KS3	−0.05 (–0.20 to 0.50)	0.500	−0.06 (–0.24 to 0.12)	0.538	−0.12 (–0.33 to 0.09)	0.252	−0.13 (–0.34 to 0.09)	0.245
KS3 to KS4	0.14 (–0.04 to 0.32)	0.121	0.11 (–0.12 to 0.33)	0.365	0.18 (–0.08 to 0.44)	0.188	0.18 (–0.08 to 0.45)	0.169

Measures are mean differences (95% CI) in the average KS decile and in the change seen over and between the four measures.

*Social factors: maternal age, socioeconomic group and education and ethnicity.

†Antenatal factors: gender, parity, weight, length and head circumference at birth.

‡Intrapartum factors: mode of delivery and maternal hypertension.

KS, Key Stage.

## Discussion

In this study, we have shown that while early educational measures are correlated with later measures for all children, the trajectory of educational measures in preterm infants varies compared with that of their term peers, but that prediction of their final outcome remains difficult. The data presented here indicate that most of the differences in trajectory seem to occur in the first few years at school, suggesting that preterm infants demonstrate some evidence of ‘catch up’ during the first few years at school, after which they appear to have similar educational trajectories to their peers.

One of the strengths of this work is that it is based on a population cohort study which prospectively collected data on many important covariates. In keeping with many cohort studies a degree of missing data is present, with around 14% of eligible infants excluded due to a lack of outcome data. We used a multiple imputation technique to reduce the impact of missing confounders, but potential selection bias needs to be considered when interpreting the results presented here. It should also be noted that this cohort is based on preterm infants born more than 20 years ago and that some changes to the educational processes, and admission policies, are likely to have occurred during this time. However, these children born preterm demonstrated similar lower scores at school as those in more recent publications.^13 14^

This work suggests, like other,^4^ that preterm infants continued to perform below their peers throughout their educational journey. However, their profile of attainment may be different, and some ‘catch up’ before the age of 11 seemed to occur. In contrast, a recent study of cognitive trajectories in extremely preterm infants was unable to find evidence of ‘catch up’, suggesting that the mechanisms here may be dependent on components other than purely cognitive skills.^7^ Our main results included a wider range of preterm birth, although subgroup analysis in this work seemed to suggest compatible if less precise results to the overall analysis. We have previously shown that educating children born preterm in their correct school year for their expected birth date (rather than their actual date) may be a cost-effective way of supporting these children.^1 6^ This work further suggests that preterm infants may need special consideration during their education and indeed may be particular sensitive to supportive interventions.^15 16^ If replicated, this work supports the idea that early support may be differentially beneficial to ex-preterm infants in optimising their development.

The reduction in correlation between early measures and later ones for preterm infants (compared with term infants) may be due to a number of factors, including simple attenuation over time or increased mortality in a subset of very disabled children. While the educational journey of these infants may change because of the early low scores (eg, more support in the classroom), unless this intervention is differential on their preterm status (ie, more or less support is put in place because of their preterm birth) then the results would still appear to remain valid. Overall, we found little to suggest that the different educational trajectories were explained by special educational needs support.

While similar results were seen in the unadjusted and adjusted results, it may be the univariable results that are perhaps most relevant, as these are the results reviewed and assessed by teachers and parents. However, even without specifically targeted interventions, this work suggests that parents and teachers should be more optimistic about the final educational outcome with preterm infants, even when early measures would suggest otherwise.

## Conclusions

The results in this work suggest that preterm infants demonstrate some evidence of ‘catch up’ during the first few years at school, with a closing of the gap in low scores, and better prediction of their final score once they have reached KS2. Premature infants appear to have similar trajectories to their peers after this point. This further emphasises the importance of early schooling and environment in these infants and suggests that support, long after their premature birth, may have additional benefits.
