# Supplementary material for: Prediction of school outcome after preterm birth: a cohort study
Source: Arch Dis Child. 2018 Oct 8;104(4):348–53. doi: 10.1136/archdischild-2018-315441 (PMC6530075; doi:10.1136/archdischild-2018-315441)

Appendix

Graphs showing i) The proportion of children scoring each individual score and overlaid with ii) the range of scores, and mean score for each derived decile. Repeated for Key Stage 1-4.

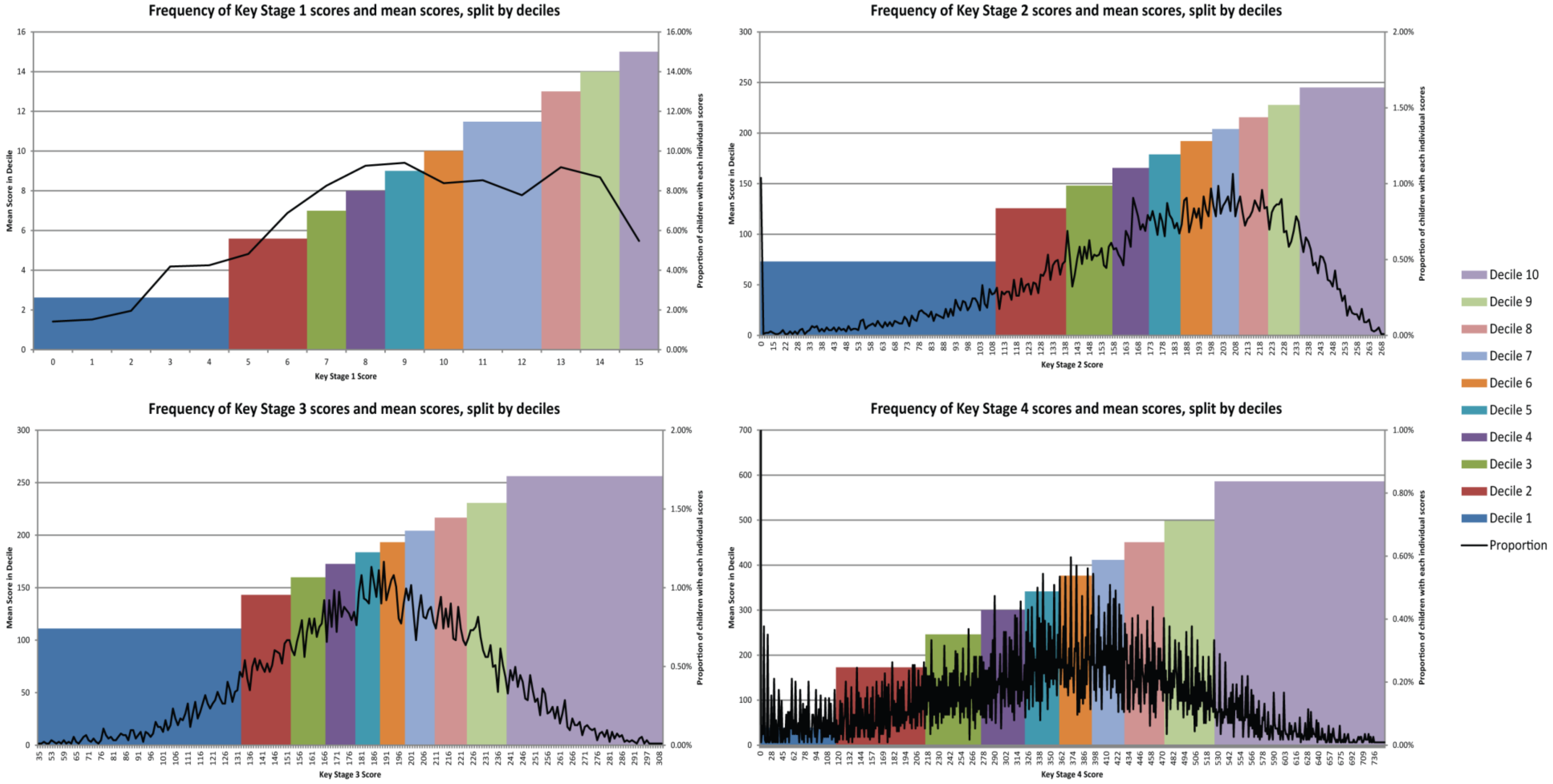

Supplement: Supplementary file 1 [file archdischild-2018-315441supp001.pdf]
